# Detection of *Plasmodium* in faeces of the New World primate *Alouatta clamitans*

**DOI:** 10.1590/0074-02760160222

**Published:** 2016-08-25

**Authors:** Gabriela Maíra Pereira de Assis, Denise Anete Madureira de Alvarenga, Daniela Camargos Costa, Júlio César de Souza, Zelinda Maria Braga Hirano, Flora Satiko Kano, Taís Nóbrega de Sousa, Cristiana Ferreira Alves de Brito

**Affiliations:** 1 Fundação Oswaldo Cruz, Centro de Pesquisas René Rachou, Laboratório de Malária, Belo Horizonte, MG, Brasil; 2 Universidade Regional de Blumenau, Blumenau, SC, Brasil; 3 Centro de Pesquisas Biológicas de Indaial, Indaial, SC, Brasil

**Keywords:** New World primates, Alouatta clamitans, simian malaria, Plasmodium simium, Plasmodium vivax, non-invasive sampling

## Abstract

*Plasmodium falciparum* and *Plasmodium vivax* have evolved with host switches between non-human primates (NHPs) and humans. Studies on the infection dynamics of *Plasmodium* species in NHPs will improve our understanding of the evolution of these parasites; however, such studies are hampered by the difficulty of handling animals in the field. The aim of this study was to detect genomic DNA of *Plasmodium* species from the faeces of New World monkeys. Faecal samples from 23 *Alouatta clamitans* from the Centre for Biological Research of Indaial (Santa Catarina, Brazil) were collected. Extracted DNA from faecal samples was used for molecular diagnosis of malaria by nested polymerase chain reaction. One natural infection with *Plasmodium simium* was identified by amplification of DNA extracted from the faeces of *A. clamitans*. Extracted DNA from a captive NHP was also used for parasite genotyping. The detection limit of the technique was evaluated in vitro using an artificial mixture of cultured *P. falciparum* in NHP faeces and determined to be 6.5 parasites/µL. Faecal samples of New World primates can be used to detect malaria infections in field surveys and also to monitor the genetic variability of parasites and dynamics of infection.

Malaria is a mosquito-borne disease caused by parasites of the genus *Plasmodium* that infect mammals, reptiles and birds. *Plasmodium* species that infect non-human primates (NHPs) are of great interest because they may be transmitted, naturally or accidentally, to humans (Deane et al. 1966, Arruda et al. 1989, Singh et al. 2004). Human infections with *Plasmodium knowlesi*, a parasite that naturally infects Old World monkeys (mainly *Macaca nemestrina* and *Macaca fascicularis*), have reinforced the thought that the proximity of humans to forests allows diseases that circulate naturally among wild animals to also occur in humans (White 2008). Malaria caused by *P. knowlesi* in humans is highly virulent and often has a fatal outcome (Galinski & Barnwell 2009). Infections with this pathogen have been recently reported in many countries of Southeast Asia (Yusof et al. 2014).

In addition to *P. knowlesi*, there are several other species of *Plasmodium* that infect NHPs: *Plasmodium cynomolgi* (Mayer, 1907), *Plasmodium inui* (Von Halberstaedter & Prowazek, 1907) and *Plasmodium schwetzi* (Warrell, 2002), among others. Two parasite species are responsible for simian malaria in the forests of Central and South America: *Plasmodium brasilianum* (Gonder & Berenberg-Gossler, 1908) and *Plasmodium simium* (Fonseca, 1939). Importantly, both *P. brasilianum* and *P. simium* can infect humans (Deane et al. 1966, Arruda et al. 1989, Cerutti-Junior et al. 2007). These parasites are morphologically, genetically and immunologically similar to the human parasites *Plasmodium malariae* and *Plasmodium vivax*, respectively (Deane 1992, Leclerc et al. 2004, Cerutti-Junior et al. 2007). Such similarities gave rise to the hypothesis that monkeys may act as a reservoir for human malaria in specific areas (Coatney 1971, Fandeur et al. 2000, Duarte et al. 2008). *P. brasilianum* is widely distributed in Central and South America. It has been found in Brazil, Colombia, Venezuela, Panama and Peru. This species naturally infects various New World monkey species, including those from the families: Aotidae, Atelidae, Callitrichidae, Cebidae and Pitheciidae (Deane 1992, Lourenço-de-Oliveira & Deane 1995, Araújo et al. 2013). In Brazil, except for the arid portions of the northeast and savannah in the southeast, the geographic range of *P. brasilianum* includes all regions and overlaps that of *P. simium*, which is restricted to the Atlantic Forest in the south and southeast (Deane 1992). In contrast to *P. brasilianum*, *P. simium* has been detected only in *Alouatta* (howler monkeys), *Brachyteles* (woolly spider monkeys) and, more recently, *Cebus* and *Sapajus* (capuchin monkeys) (Deane 1992, Duarte et al. 2008, Alvarenga et al. 2015).

Therefore, molecular studies of simian *Plasmodium* species are indispensable to understanding the true prevalence, transmission dynamics, and diversity of this parasite, as well as to elucidate the evolutionary history of *Plasmodium* species in the New World. However, the use of blood samples for both optical microscopy and for diagnostics based on molecular techniques is hampered by the need to capture and handle wild animals in the field, which is not feasible in many situations. Therefore, DNA extracted from samples that are collected using a simple, less invasive and inexpensive method can facilitate a better understanding of simian malaria, as well as provide information for phylogenetic analyses of these parasites. Recent studies have shown that saliva, urine and faeces from malaria patients contain trace amounts of *Plasmodium* DNA that can be amplified by polymerase chain reaction (PCR) and, therefore, can be used as alternative specimens for epidemiological surveys (Nwakanma et al. 2009, Jirků et al. 2012). For phylogenetic studies of *Plasmodium*, DNA sequences have been obtained from faecal samples of Old World monkeys. A recent study elucidated the origin of *P. falciparum* and its reservoir using this approach (Liu et al. 2010). Another study conducted by Liu and colleagues (Liu et al. 2014) indicated that all strains of *P. vivax* are derived from a single ancestor that escaped out of Africa. This study was based on DNA extractions from chimpanzee and gorilla faeces from Africa. In 2015, Siregar and colleagues (Siregar et al. 2015) optimised protocols for the non-invasive sampling and isolation of malaria parasites from naturally infected Old World monkeys. However, despite the successful molecular diagnosis of malaria from faeces of Old World monkeys, this methodology has not been standardised for New World monkeys.

## MATERIALS AND METHODS

*Monkey faecal sample collection* - For this study, faeces were collected from captive NHPs from the Centre for Biological Research of Indaial (CEPESBI), Santa Catarina, Brazil. CEPESBI (IBAMA register number 1/42/98/000708-90) is a unit for wild monkey protection, located in the Valley of Itajaí (26º53’52” S/49º13’54” W) in the Atlantic Forest. Faecal samples from *Alouatta guariba clamitans* (n = 23) were stored in RNAlater (Qiagen) at a ratio of 1:2 and transferred to -20ºC within 24 h, where they were held until DNA was extracted.

*DNA extraction from faecal samples* - First, the faeces in RNAlater were placed in gauze to remove any leaves and seeds. DNA was extracted from faecal samples using a modified protocol for the QIAamp DNA Mini Stool Kit (Qiagen). To lyse cells, 500 µL of a faecal sample in RNAlater was homogenised in 5 mL of ASL buffer and vortexed. This suspension was heated to 70ºC for 5 min, vortexed for 15 more sec, and centrifuged for 1 min at 20,000 × *g* to obtain aggregate faecal particles. The supernatant (1.2 mL) was transferred to a new 2-mL microcentrifuge tube, and the pellet was discarded. Then, half of an InhibitEX tablet was added to each sample, and the sample was vortexed immediately for 1 min or until the tablet was completely dissolved. The resulting suspension was centrifuged at 20,000 × *g* for 3 min. For the rest of the procedures, the manufacturer´s instructions were followed. To elute the DNA, 100 µL of Buffer AE was added, incubated for 5 min at room temperature (20-24ºC), and centrifuged at 20,000 × *g* for 1 min. Finally, the DNA was stored at -20ºC. Filter pipette tips were used in all stages of sample processing to prevent cross-contamination.

*Artificial mixture with cultured P. falciparum* - One hundred microliters of cultured *P. falciparum* strain 3D7 (parasitaemia of 6.5 × 10^5^ parasites/µL) was added to 400 µL of uninfected brown howler monkey stool (in RNAlater, 1:2). The same procedure was performed with cultured *P. falciparum* strain W2 (parasitaemia of 5.0 × 10^5^ parasites/µL). DNA was extracted from these artificial mixtures using a QIAamp DNA Stool Mini Kit (Qiagen) as described above. DNA obtained from *P. falciparum* 3D7 mixed with faeces was 10-fold diluted from 1:100 to 1:1,000,000. These dilutions were tested by nested PCR to diagnose malaria (Snounou et al. 1993).

*PCR of the mammalian cytochrome B gene* - To assess the quality of DNA from samples, the mammalian cytochrome B gene was amplified by PCR. The protocol described by Kocher et al. (1989) was used with modifications. A fragment of 350 bp was amplified from a reaction volume of 20 µL containing 50-100 ng of DNA, 0.625 mM each primer (Table I), 0.125 mM each dNTP, 1.875 mM MgCl_2_, 1 U Taq DNA polymerase (Invitrogen, California, USA), and the enzyme buffer provided by the manufacturer. The reactions were carried out in a Mastercycler personal thermocycler (Eppendorf). The cycling conditions included one step of 95ºC for 5 min, followed by 35 cycles at 95ºC for 30 sec, 58ºC for 30 sec, and 72ºC for 1 min, and ended in an extension step at 72ºC for 5 min and 4ºC until the products were collected.

**TABLE I:** Variants of polymerase chain reaction (PCR) targets, primer sequences, sizes of the amplified fragments and references for the PCR protocols used in this study

PCR	Target	Primers	Sequence (5’ - 3’)	Fragment	References
Nested 1 Reaction	18SSU rRNA gene of *Plasmodium* sp.	rPLU5 rPLU6	5’CCTGTTGTTGCCTTAAACTTC 3’ 5’TTAAAATTGTTGCAGTTAAAACG3’	1200pb	Snounou et al. (1993)
Nested 2 Reaction	18SSU rRNA gene of *P. vivax*, *P. falciparum* and *P. malariae*	rVIV1 rVIV2 rFAL1 rFAL2 rMAL1 rMAL2	5’CGCTTCTAGCTTAATCCACATAACTGATAC3’ 5’ ACTTCCAAGCCGAAGCAAAGAAAGTCCTTA 3’ 5’TTAAACTGGTTTGGGAAAACCAAATATATT3 5’ACACAATGAACTTCAATCATGACTACCCGTC3’ 5’ATAACATAGTTGTACGTTAAGAATACCGC 3’ 5’AAATTCCCATGCATAAAAAATTATACAAA3’	120 pb 205 pb 144 pb	Snounou et al. (1993) Snounou et al. (1993) Snounou et al. (1993)
PCR conventional	*Pvms1bl10* gene of *P. vivax*	MSP1bl10F MSP1bl10R	5’ CAAGCCTACCAAGAATTGATCCCCAA 3’ 5’ATTACTTTGTCGTAGTCCTCGGCGTAGTCC 3’	200 pb	de Araújo et al. (2012)
PCR conventional	*Pvmsp1bl2* gene of *P. vivax*	MSP1bl2F MSP1bl2R	5’ GACGATATTGGAAAATTGGA 3’ 5’CTCCTTCAGCACTTTCACGCGCTT 3’	400 pb	de Araujo et al. (2012)
PCR conventional	Mammalian cythocrome B	cyBF cyBR	5’ CCCCTCAGAATGATATTTGTCCTCA 3’ 5’ CCATCCAACATCTCAGCATGATGAAA 3’	350 pb	Kocher et al. (1989)
PvMS1	Microsatellite	MS1F MS1R	5’ CTATCTGAGGAATGGGGA3’ 5’ATTTACTATGACGAAGGTGA3’	Variant	Rezende et al. (2010)
PvMS5	Microsatellite	MS5F MS5R	5’TGCTATTTGCTCGTCTGT3’ 5’GAGCGTTATCATCATTAG3’	Variant	Rezende et al. (2010)
PvMS6	Microsatellite	MS6F MS6R	5’ACACATTTGACACAGTTCC3’ 5’ATGCCCTGGTCCCTACAA3’	Variant	Rezende et al. (2010)
PvMS7	Microsatellite	MS7F MS7R	5’GTATTCCCCGTCTTGTCC3’ 5’CTTTGTCCGTTCTTATTTCT3’	Variant	Rezende et al. (2010)

*Molecular diagnosis of Plasmodium infection* - The samples were subjected to nested PCR, as described by Snounou et al. (1993), using primers to detect human *Plasmodium* species (*P. falciparum*, *P. vivax* and *P. malariae*) by targeting the small subunit (SSU) 18S of the ribosomal RNA (18S rRNA) gene (Table I). Briefly, all PCR reactions were performed in 20-μL volumes containing 250 μM each primer, 10 μL of Master Mix (Promega) (0.3 units of Taq polymerase, 200 μM each DNTP, and 1.5 mM MgCl_2_), and 0.8 μL (~100-200 ng) of DNA. Bovine serum albumin (BSA) was added to the PCR mixture at a final concentration of 0.1 μg/μL. PCR assays were performed using an automatic thermocycler (PTC-100TM v. 7.0) (MJ Research, Inc., USA) with the following cycling parameters: an initial denaturation step at 95ºC for 5 min, followed by 24 cycles of 58ºC for 2 min, 72ºC for 2 min, and 94ºC for 1 min, and one final step at 58ºC for 2 min, an extension at 72ºC for 5 min, and then 4ºC until the products were collected. The cycling parameters for the second round of PCR were the same as the first reaction, except for the use of 29 cycles for amplification.

To prevent cross-contamination, the DNA extracts and PCR preparations were performed in “parasite DNA-free rooms” that were distinct from each other. Furthermore, each of these separate areas had different sets of pipettes, and all procedures were performed using pipette tips with aerosol barriers. DNA extraction was performed twice on different days. Every PCR reaction had a negative control, in which DNA has replaced by water, as well as positive controls for each pair of primers. The sources of genomic DNA used as positive controls in the nested PCR were *P. falciparum* strain 3D7 maintained in the Laboratory of Malaria (René Rachou Research Centre - CPqRR, FIOCRUZ, Belo Horizonte, Minas Gerais, Brazil), a patient with a high *P. vivax* parasitaemia confirmed by Giemsa-stained blood smears, and a plasmid containing DNA from *P. malariae* (MRA-179 *P. malariae* small subunit rRNA Nest 1 PCR Plasmid Clone 34-MR4). The amplified fragments were resolved by electrophoresis on 2% agarose gel dissolved in TAE buffer (40 mM Tris-acetate, 1 mM EDTA) in a horizontal system (Bio-Rad) at 100 V for approximately 30 min. Gels were stained with 5 μg/mL ethidium bromide (Invitrogen) and examined under a UV transilluminator (BioDoc-It system), and images were captured using a digital system. Electrophoresis was performed in a room specifically for use with amplified DNA, with dedicated sets of pipettes and pipette tips with barriers.

*Genotyping of molecular markers* - Six molecular markers were selected for this study: blocks 2 and 10 of the merozoite surface protein-1 gene of *P. vivax* (*Pvmsp1B2* and *Pvmsp1B10*) and four *P. vivax* microsatellites (MSs; *PvMS1*, *PvMS5*, *PvMS6* and *PvMS7*). These fragments were amplified using specific primers and previously described conditions with minor modifications (Rezende et al. 2010, de Araújo et al. 2012). All forward primers for genotyping were conjugated to the fluorescent dye 6-FAM. Nested PCR was used to amplify both *Pvmsp1* blocks (BL2 and BL10). The first reaction was conducted in a volume of 10 μL using 100-200 ng DNA, 0.5 μM each primer (Integrated DNA Technologies, San Diego, CA, USA) (Table I), 0.125 mM dNTP mix, 0.75 mM MgCl_2_, 1 U Taq DNA polymerase (Invitrogen, California, USA), and appropriate enzyme buffer. The amplifications were performed on a Veriti thermocycler (Applied Biosystems, Carlsbad, CA, USA), using the following cycling conditions: 94ºC for 4 min; 30 cycles of 94ºC for 1 min, then 55ºC for 1 min for *Pvmsp1b2* and 63ºC for 1 min for *Pvmsp1b10*, and 72ºC for thirty seconds; followed by a final extension step at 72ºC for 5 min and 4ºC indefinitely. The following mixture was subjected to nested PCR in a 20-μL volume: 1 μL of product from the first PCR reaction (diluted 1:500 in H_2_O for *Pvmsp1b2* and 1:50 in H_2_O for *Pvmsp1b10*), 0.5 μM each primer (Integrated DNA Technologies, San Diego, CA, USA), 0.125 mM dNTP mix, 3 mM MgCl_2_, 1 U recombinant Taq polymerase (Invitrogen), and 2 μL 10× Taq polymerase buffer (Invitrogen, Carlsbad, CA, USA). The cycling parameters included the following: 1 cycle at 94ºC for 4 min; 25 cycles consisting of 94ºC for 40 sec, 63ºC for 1 min for *Pvmsp1b2* and 60ºC for 1 min for *Pvmsp1b10*, and 72ºC for 40 sec; and a final cycle of 10 min at 72ºC.

The MSs (*PvMS1, PvMS5, PvMS6*, and *PvMS7*) were amplified in a 20-µL volume of 100-200 ng DNA, 1 µM each primer (Table I), 0.125 mM dNTP mix, 1.5 mM MgCl_2,_ 1 U Taq DNA polymerase (Invitrogen, Carlsbad, CA, USA), and 2 μL 10× Taq polymerase buffer (Invitrogen, Carlsbad, CA, USA). The amplifications were performed in a Veriti thermocycler (Applied Biosystems, Carlsbad, CA, USA) with one cycle of 94ºC for 2 min; 35 cycles at 94ºC for 30 sec, and then 53.4ºC for MS1, 56ºC for MS5 and MS7, and 58.6ºC for MS6, all for 20 sec; and 72ºC for 30 sec, ending in an extension step at 72ºC for 2 min.

For capillary electrophoresis, 2 μL of a PCR product was mixed with 0.25 µL of the standard size ET550-Rox genotyping size standard (GE Healthcare) and 7.75 µL of 0.1% formamide HI-DITM. After capillary electrophoresis, alleles were visualised and scored using an automated DNA sequencer (ABI 3730xl DNA Analyzer, Applied Biosystems, California, USA). Their lengths and relative abundance (peak heights in electropherograms) were determined using GeneMapper Software version 4.1 (Applied Biosystems, California, USA). For the electropherogram analysis, the minimum peak height was set to 150 arbitrary fluorescence units (rFU). Additionally, a cut-off value for minor peak detection of one-third the height of the predominant peak was used to exclude artefact peaks.

## RESULTS AND DISCUSSION

**Fig. 1:**
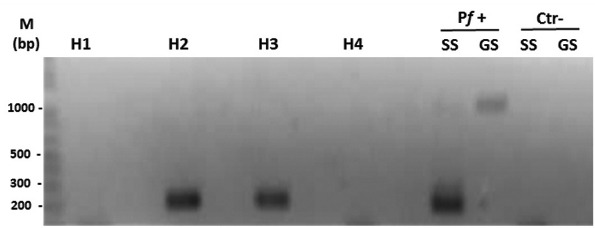
: nested polymerase chain reaction used to amplify the 18SSU rRNA fragment of *Plasmodium falciparum* in an artificial mixture of the parasite with uninfected faeces from a New World primate. Results were visualized on a 2% agarose gel stained with ethidium bromide. M: molecular weight marker (1-kb DNA ladder); H1 and H4: faecal samples without the parasite included; H2 and H3: faecal samples with *P. falciparum* strains 3D7 and W2, respectively; Pf+: positive control for *P. falciparum* (cultured) using genus-specific primers (GS, 1,000 bp) and species-specific (SS, 240 bp) primers; Ctr-: negative control (without DNA).

Two aliquots of a stool sample from an uninfected captive southern brown howler monkey were mixed with 100 µL of blood from cultured *P. falciparum* strain 3D7 (13% parasitaemia) or W2 (10% parasitaemia). Another two aliquots were used as a negative control without the addition of *Plasmodium*. These four aliquots were used for genomic DNA extractions, and DNA of the 18SSU rRNA gene was amplified with primers specific for *P. falciparum* (Fig. 1). A fragment of 205 bp was successfully amplified from DNA extracted from the artificial mixture of monkey faeces with cultured *P. falciparum* and from a positive control of *P. falciparum* DNA directly extracted from culture.

**Fig. 2:**
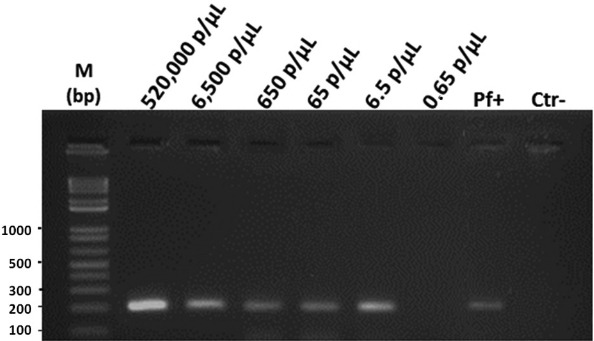
: detection limit of the parasite by polymerase chain reaction (PCR) in an artificial mixture of uninfected faeces from a New World primate with *Plasmodium falciparum* from in vitro culture. Molecular diagnosis of *Plasmodium* infection by nested PCR using primers for *P. falciparum* (205 bp)*.* Results shown in a 2% agarose gel stained with ethidium bromide. M: molecular weight marker (1-kb DNA ladder); Pf+: positive control for *P. falciparum*, Ctr-: negative control (without DNA).

In order to determine the detection limit of this assay, DNA from the artificial mixture of simian stool with cultured *P. falciparum* 3D7 was used in different concentrations. The DNA extracted from the artificial mixture of monkey stool (uninfected) and *P. falciparum* culture (6.5 × 10^5^ parasites/µL) and serial 10-fold dilutions from 1:100 to 1:1,000,000 (equivalent amounts of parasites shown in Fig. 2) were used to detect *Plasmodium* infection by nested PCR (Fig. 2). The detection limit of this protocol was 6.5 parasites/µL (dilution 1:100,000). Only the last DNA dilution, corresponding to 0.65 parasites/µL, was negative by the PCR assay.

**Fig. 3:**
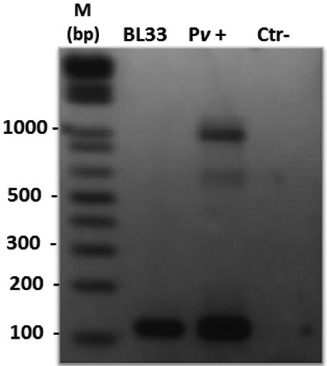
: natural *Plasmodium vivax*/*Plasmodium simium* infection detected in DNA extracted from the faeces of a captive *Alouatta clamitans* (BL33)*.* Molecular diagnosis of *Plasmodium* infection was accomplished by nested polymerase chain reaction (18SSU rRNA) using specific primers for *P. vivax*. Results shown in a 2% agarose gel stained with ethidium bromide. Pv+: positive control (DNA from a *P. vivax*-infected patient) and Ctr-: negative control (without DNA); M: molecular weight marker (1-kb DNA ladder).

Faecal samples from 23 brown howler monkeys, *A. clamitans*, from the Centre for Biological Research of Indaial (Santa Catarina, Brazil) were used for DNA extraction. The mammalian cytochrome b gene was amplified to assess the quality of DNA in all samples (data not shown). Molecular diagnosis of *Plasmodium* infection was accomplished by nested PCR (18SSU rRNA) from the DNA extracted from faeces. One natural *P. simium*/*P. vivax* (BL33) infection was detected (Fig. 3). Negative controls did not show any amplified fragments, whereas positive controls showed fragments of the expected sizes (Fig. 3).

Surveys to determine the prevalence of *Plasmodium* parasites in monkeys are limited, primarily because of the difficulties involved in catching and handling animals in the field and the high cost of capture. Moreover, because of low parasitaemias and the chronicity of infection associated with several neotropical *Plasmodium* species, malaria is notoriously difficult to detect in monkeys by microscopy (Coatney 1971). PCR methods are considered more sensitive and specific; consequently, large-scale surveys to assess the prevalence and distribution of *Plasmodium* infections in NHPs that are based on PCR are required. Validation of a biological sampling technique that allows genomic DNA extraction without the necessity of monkey capture would be useful for epidemiological surveys of simian malaria in New World monkeys. Here, we report the first study using faecal samples of New World monkeys to detect neotropical *Plasmodium* species. In addition, the present study is the first to detect a natural infection with *P. simium* using DNA extracted from the faeces of a captive *A. clamitans* in southern Brazil.

Faeces are one of the most difficult biological samples to use for diagnosis by PCR because of the presence of various bacteria and products of feeding such as complex vegetal polysaccharides, which are potential inhibitors of the amplification (Monteiro et al. 1997). These challenges necessitate the use of a robust extraction and amplification methods. PCR inhibitors were removed during the extraction with InhibitEX resin and an optimised buffer in the silica membrane-based purification kit (QIAamp DNA Stool Mini Kit, Qiagen) (Fitzpatrick et al. 2010). We also tested the QIAamp Fast DNA Stool Mini Kit (Qiagen), because InhibitEX was easier to add as a buffer than as a tablet. However, no amplification was observed using this DNA isolation kit (data not shown).

In order to ensure DNA stability*,* monkey faeces were maintained in RNA Later at room temperature (20-24ºC) and frozen as soon as possible. Moreover, to overcome the effects of inhibitors that were not eliminated during the extraction step, we also used BSA as an additive during amplification.

Mharakurwa and colleagues (Mharakurwa et al. 2006) showed that shorter PCR amplicons could be amplified effectively from parasite DNA in human urine and saliva, despite the fact that DNA may be highly fragmented by the time it reaches these fluids. To optimise the detection of parasite DNA at low concentrations in faeces, it is necessary to use a multicopy target gene and consider the PCR amplicon size (Siregar et al. 2015). In this study, the MS and polymorphic blocks of the MSP1 gene, all of them resulting in short amplicons, were used for parasite genotyping. As measures to avoid contamination were undertaken and the PCR reactions were very well controlled (see Methods), the results presented here are not believed to be a result of cross-contamination in the laboratory.

Recently, the detection of DNA in faeces of monkeys by PCR was used to indicate the presence of parasites in the red blood cells of a host, rather than the presence of pre-erythrocytic parasites not established in the host (Abkallo et al. 2014). Here, the amplification of different genomic targets contradicts this; however, this possibility could not be excluded.

To confirm natural *Plasmodium* infection, parasites in stool sample BL33 were genotyped using four MS loci and two polymorphic regions of MSP1 (Table II). The sizes of fragments obtained were compared to those of other monkeys genotyped in the laboratory and to human samples infected with *P. vivax* (Rezende et al. 2010). Based on results from MS1, MS5 and MS6, alleles previously identified in humans infected with *P. vivax* were also found in this study (Rezende et al. 2010, de Araújo et al. 2012). Furthermore, MS7 and the polymorphic regions of MSP1 in *P. vivax* (blocks 2 and 10) were found in both humans and monkeys infected with *P. simium* in a study conducted in other areas of the Atlantic Forest (unpublished data), suggesting that similar parasites are circulating among humans from endemic areas and monkeys from the Atlantic Forest. However, additional samples and molecular markers are needed to confirm this hypothesis. Results of the present study indicate that it is possible to detect *Plasmodium* in the faeces of New World monkeys and to use the data to monitor the genetic variation and dynamics of infection with the parasite.

**TABLE II:** Genotyping of *Plasmodium simium* using molecular markers

Molecular Marker	Fragment size (pb)
MS1	224*
MS5	191***
MS6	209***
MS7	324**
MSP1 (BLOCK 2)	397***
MSP1 (BLOCK 10)	374**

*: fragment size previously obtained from human samples; **: fragment size previously obtained from monkey samples; ***: fragment size previously obtained from monkey and human samples.

According to the National Malaria Control Program (PNCM), Secretary for Health Surveillance (SVS), Ministry of Health, from January 2006 to December 2013, 8,410 cases of malaria were reported in the extra-Amazon Region, and 1,068 of these cases were autochthonous. More specifically, in 2013, of the 827 extra-Amazonian cases, 10.6% (88 cases) was acquired from autochthonous transmission (de Pina-Costa et al. 2014). In south and southeastern Brazil, human malaria transmission has been virtually eliminated or, for the past four decades, has only been reported during scattered outbreaks that began with imported cases (de Pina-Costa et al. 2014). However, a few autochthonous human malaria cases have been detected in the Atlantic Forest region, particularly in the mountain valleys in Southeast Brazil (Miguel et al. 2014). In such an environment, malaria transmission is supported by the bromeliad mosquito *Anopheles (Kerteszia) cruzii*, which has been implicated as the natural vector for both human and simian malaria in South and Southeast Brazil (Deane 1992). Therefore, in the 1990s, Deane and other authors more recently have suggested that human malaria in the Atlantic Forest could be a zoonosis with non-human primates NHPs as the reservoirs (Deane 1992, Duarte et al. 2008, de Pina-Costa et al. 2014). This study identified a natural *P. simium* infection by DNA extraction from the stool of a captive New World monkey. Taking into account that faeces collected from captive monkeys were probably relatively fresh, this protocol for field-collected samples still needs to be validated under more adverse environmental conditions. However, DNA has been successfully obtained from monkey’s faeces in the field in Africa (Liu et al. 2010). Once autochthonous human cases of malaria are reported, the presence of wild reservoirs may have important implications for public health because of the close contact between humans and monkeys in some parts of the Atlantic Forest (de Pina-Costa et al. 2014).

Cases of simian malaria caused by neotropical *Plasmodium* species are neglected. The question of whether these NHPs are reservoirs for human malaria might become important, especially if the disease is eradicated or eliminated in human populations. Epidemiological surveys of *Plasmodium* infections in wild New World monkeys are needed to assess the risk of exposure to zoonotic malaria, as recently described for *P. knowlesi*. Validation of a genomic DNA extraction method based on simple, less invasive and inexpensive sample collections can contribute to a better understanding of simian malaria. Faecal samples, therefore, offer an attractive alternative specimen for the detection of primate malaria. This is the first study to detect neotropical *Plasmodium* species from the faeces of New World monkeys. The present study identified a natural *P. simium* infection based on DNA extracted from the faeces of a captive *A. clamitans* (BL33) in southern Brazil.
